# Navigating the Esophageal Enigma: A Vascular Odyssey of Dysphagia

**DOI:** 10.7759/cureus.37993

**Published:** 2023-04-22

**Authors:** Olayiwola Bolaji, Faizal Ouedraogo, Olanrewaju K Adabale, Onyinye S Ugoala

**Affiliations:** 1 Internal Medicine, University of Maryland Capital Region Medical Center, Largo, USA; 2 Medicine, University of Maryland Capital Region Health, Largo, USA; 3 Internal Medicine, East Carolina University, Greenville, USA; 4 Internal Medicine, College of Medicine, University of Lagos, Lagos, NGA

**Keywords:** rare cause of dysphagia, aortic arch variation, congenital vascular anomaly, revascularization, minimally invasive surgery, esophageal obstruction, vascular compression, thoracic endovascular aortic repair (tevar), aberrant right subclavian artery (arsa), dysphagia lusoria

## Abstract

We report a 58-year-old female with severe throat pain, difficulty swallowing, choking on solid meals, coughing, and hoarseness. CT angiography of the chest revealed vascular compression of the esophagus by an aberrant right subclavian artery (ARSA). The patient underwent thoracic endovascular aortic repair (TEVAR) and revascularization to address the ARSA. The patient experienced significant improvement in her symptoms following the surgical intervention.

Dysphagia lusoria is a rare condition involving compression of the esophagus and airway by an ARSA. While medical management is the first line of treatment for mild symptoms, surgical intervention is often necessary for severe cases or those unresponsive to conservative management. TEVAR with revascularization is a feasible and minimally invasive option for treating symptomatic non-aneurysmal ARSA, potentially resulting in favorable outcomes.

## Introduction

An aberrant right subclavian artery (ARSA), also known as arteria lusoria, is the most common congenital anomaly of the aortic arch, accounting for approximately 60% of all aortic arch anomalies [1]. The incidence of ARSA ranges from 0.5-1.8%, with a prevalence of 0.16-4.4% in the general population [1,2]. Typically, the ARSA arises from the descending thoracic aorta (DTA) distal to the origin of the left subclavian artery [3]. In the majority of cases, it courses posterior to the esophagus; however, it can also lie anterior to the trachea (5%) or between the esophagus and trachea (15%) [4].

Most patients with ARSA remain asymptomatic throughout their lives [5]. However, in some cases, the ARSA's vascular compression of the esophagus may result in dysphagia, a rare clinical manifestation known as "dysphagia lusoria" [2]. Dysphagia lusoria typically presents with difficulty swallowing solids and occurs in approximately 8-10% of individuals with ARSA [4].

Management of dysphagia lusoria depends on the severity of the patient's symptoms. Patients with mild symptoms are usually managed conservatively with dietary modifications and swallowing therapy [6]. However, more severe or intractable cases may require surgical intervention, including vascular reconstruction or bypass procedures [7]. In this case report, we describe the surgical management of a patient presenting with dysphagia secondary to vascular compression by an aberrant right subclavian artery.

## Case presentation

A 58-year-old female with a medical history of hypertension, diabetes mellitus, gastroesophageal reflux disease (GERD), and peptic ulcer disease presented to the emergency department (ED) with severe throat pain, difficulty swallowing, choking on solid meals, coughing, and hoarseness. Initial vital signs showed a temperature of 37.2 °C (99.02 °F), respiratory rate of 20 breaths per minute, blood pressure of 110/64 mmHg, heart rate of 94 bpm, and oxygen saturation (SpO2) of 94%. Laboratory workup revealed a WBC of 14.1 x 10^3/µL, hematocrit level of 29.2%, hemoglobin of 9.5 g/dL, and hemoglobin A1c (HbA1c) of 6.8%.

**Figure 1:**
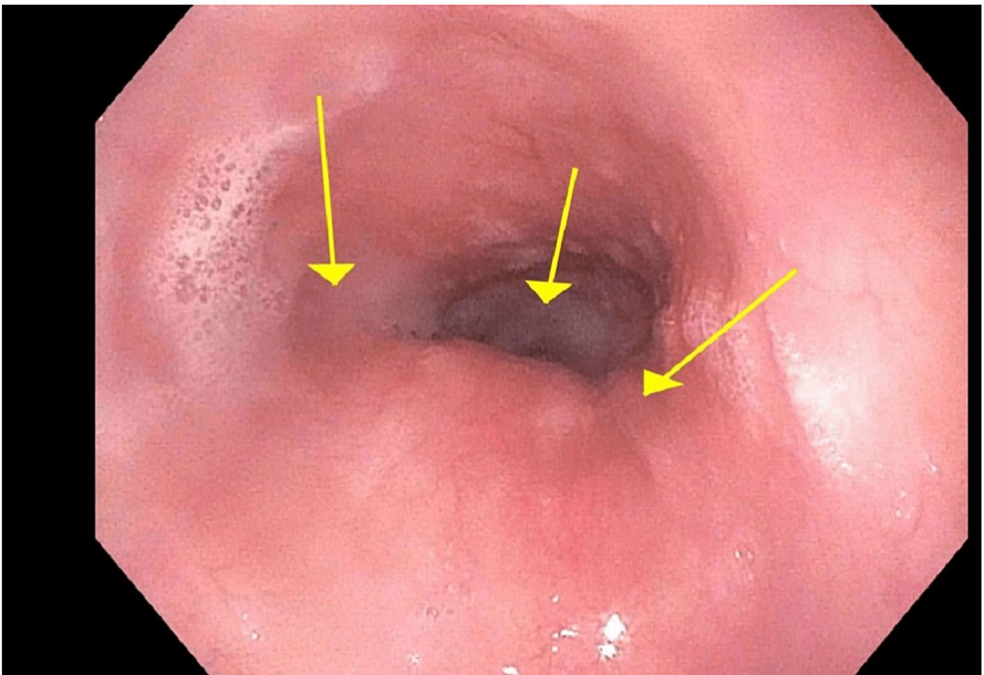
The endoscopic image displays a distinct extrinsic constriction of the esophagus, which is clearly visible at a range of 24 to 26 centimeters from the incisors. An arrow highlights the region of this extrinsic narrowing.

Physical examination demonstrated a left-sided facial droop, left upper extremity weakness, and numbness with a tingling sensation. Given the concern for stroke, a head CT scan was performed, which was unremarkable for any acute intracranial abnormalities or bleeding. Echocardiography showed an ejection fraction of 50-55% with normal left and right ventricular systolic function. Chest X-ray did not reveal any contributory findings, and esophagogastroduodenoscopy showed extrinsic compression, as shown in Figure 1.

**Figure 2:**
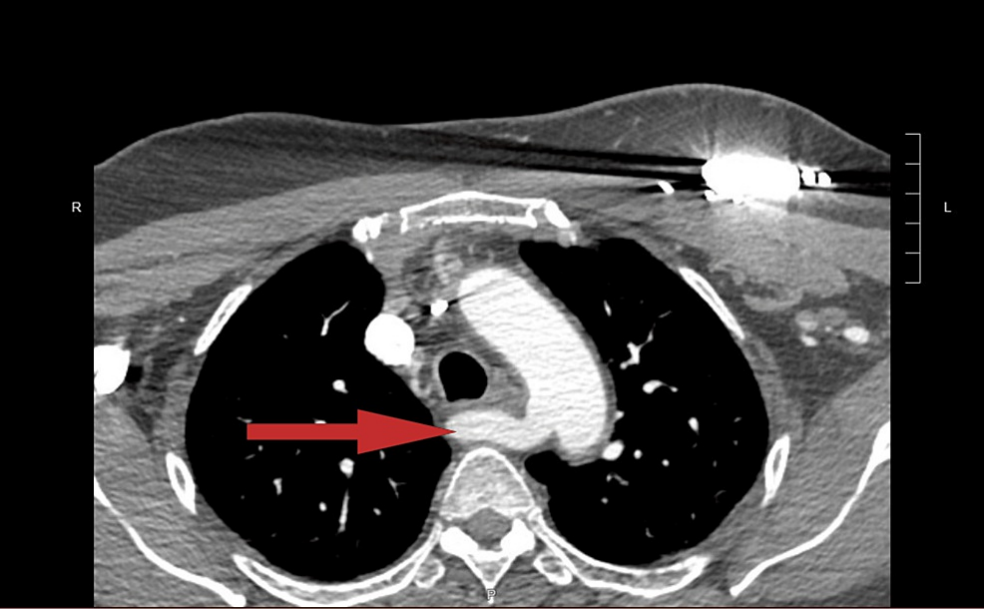
The chest CT angiography image displays an aberrant right subclavian artery (indicated by the arrow) originating from the aortic arch.

**Figure 3:**
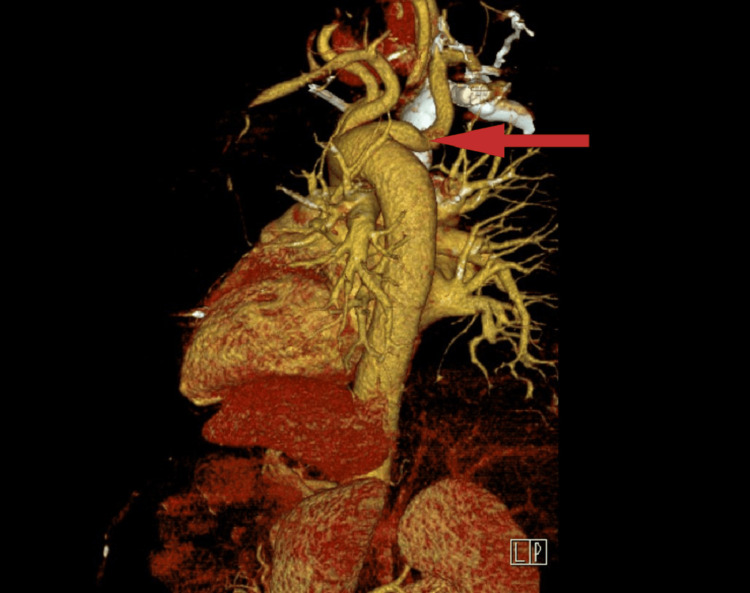
The 3D reconstruction of the chest CT angiography image visualizes an aberrant right subclavian artery (marked by the arrow) emerging from the aortic arch.

These findings prompted a CT angiography of the chest, which demonstrated vascular compression of the esophagus by an aberrant right subclavian artery arising from the descending aorta. This anomaly created an arterial ring around the esophagus and airway between the aberrant right subclavian artery and the displaced origin of the right common carotid artery, as illustrated in Figure 2 and Figure 3.

**Figure 4:**
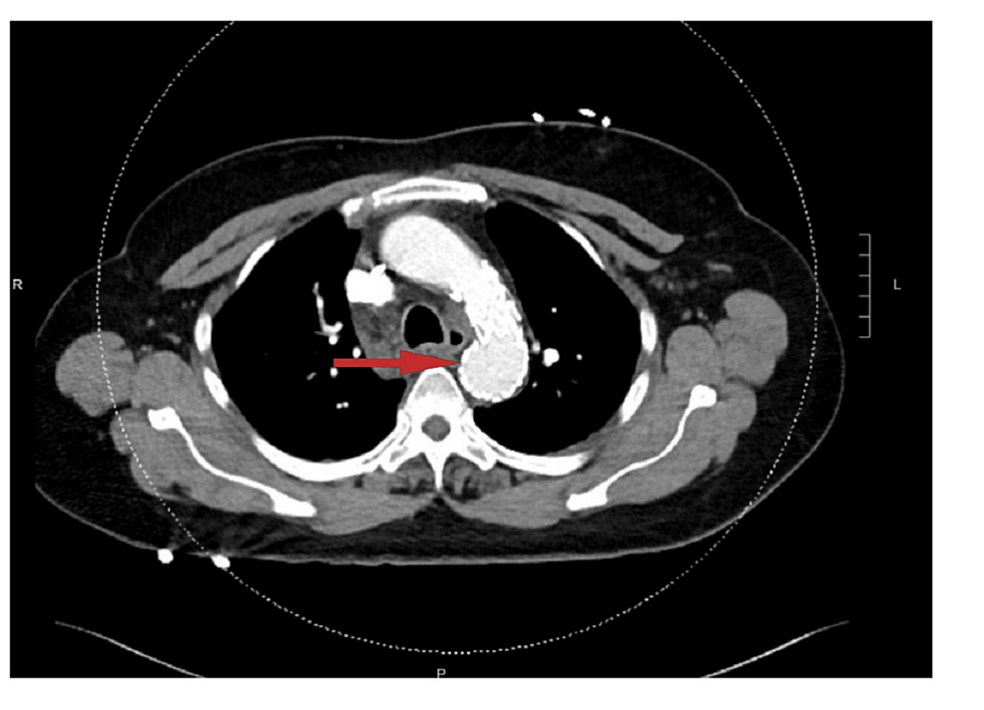
The chest CT angiography image demonstrates the aortic arch subsequent to the successful surgical correction of an aberrant right subclavian artery. The arrow indicates the absence of any residual aberrant right subclavian artery, confirming the effectiveness of the repair.

Due to the failure of medical management, vascular surgery teams were consulted. Surgical correction of the aberrant right subclavian artery involved creating a bypass through right carotid-subclavian transposition, followed by revascularization of the left upper extremity using a Gore thoracic branch endoprosthesis (TBE) and thoracic endovascular aortic repair (TEVAR) graft. Figure 4 shows images post-repair.

Subsequently, the patient underwent a right aberrant subclavian artery to right common carotid artery transposition through a right-sided supraclavicular incision and endovascular repair of ARSA using a Gore TBE TEVAR endograft performed percutaneously.

The speech and swallow team co-managed the patient with physical therapy, significantly improving her hoarseness, phonation, and dysphagia. The patient was discharged on the 12th day of admission and followed up in the outpatient clinic two weeks later, showing a remarkable improvement in her dysphagia.

## Discussion

Hunauld first reported ARSA in 1735, and Gross described the first successful surgical treatment by simple ligation and division in 1946 [8]. An aberrant right subclavian artery is a rare anomaly typically arising from the aortic arch distal to the left subclavian artery. It most frequently crosses behind the esophagus to the right axillary artery [9]. The aortic arch usually gives rise to three vessels: the brachiocephalic trunk (which divides into the right common carotid and right subclavian arteries), the left common carotid artery, and the left subclavian artery [10]. Embryologically, ARSA results from the persistence of the seventh intersegmental artery of the right dorsal aorta and a disturbance of the right fourth pharyngeal arch, which develops the innominate artery [1,11].

Studies have demonstrated an association between ARSA and trisomies, such as trisomy 18 and 21, along with other congenital anomalies that are frequently cardiac in origin [12]. ARSA is congenital; however, it usually becomes symptomatic only in the fourth to fifth decade of life, as in our patient, who presented in her fifth decade. The postulated explanation for the delay in presentation is the atherosclerotic changes in the aorta and the aberrant artery and the age-related increase in esophageal rigidity [1]. It could also result from aneurysmal dilation, termed Kommerell diverticulum (due to non-involution of the right dorsal aorta) [3]. In children, symptoms are usually due to the vessel appearing taut across the posterior esophagus. The symptoms commonly associated with dysphagia lusoria include dysphagia, dyspnea, cough, retrosternal pain, and unintentional weight loss [13]. Our patient's presenting complaints included dysphagia and cough. Asymmetric radial pulses are rare in dysphagia lusoria [1].

The diagnosis of this condition is typically established using a barium esophagogram, followed by CT angiography or magnetic resonance (MR) angiography to define the vascular anatomy [2]. An upper endoscopy can reveal a posterior pulsatile impression on the esophagus, while manometry is usually not helpful in diagnosing this condition.

The management of dysphagia lusoria primarily depends on symptom severity. Less severe cases can be treated solely with dietary modification, such as eating slower, smaller bites, and sipping liquids. Medical treatment with proton pump inhibitors (PPIs), antacids, or H2-receptor antagonists can be effective in some patients. Surgical management is predominantly for patients with more severe symptoms, including those with aneurysm formation [13].

Surgical approaches may include right anterolateral thoracotomy, right supraclavicular (as seen in our patient), and median sternotomy [13]. An et al. chose a partial median sternotomy approach because it provided adequate exposure to access both the base of the right subclavian and the right carotid artery for the end-to-side anastomosis [14], particularly in pediatric patients with other cardiac anomalies. Other authors have preferred left thoracotomy for the surgical management of dysphagia lusoria with resection of the ARSA from its origin to the right border of the esophagus [7]. However, our patient underwent minimally invasive TVAR with revascularization of the ARSA without an aneurysm. This resulted in minimal blood loss, reduced postoperative wound care, minimal scarring, and a shorter hospital stay. It is important to note that the minimally invasive approach has specific indications and limitations. It is particularly suitable for patients with symptomatic non-aneurysmal ARSA who are not adequately managed with medical therapy. However, potential risks, such as left arm ischemia and basilar artery ischemia, may arise when the left subclavian artery is close to the ARSA and the right vertebral artery is dominant. Careful preoperative planning and assessment of the patient's anatomy through imaging studies can help minimize these risks and determine the appropriateness of this approach for each patient.

## Conclusions

This case highlights the uncommon condition of dysphagia lusoria, characterized by an aberrant right subclavian artery (ARSA) causing compression of the esophagus and airway. Initial management for patients presenting with mild symptoms typically involves medical therapy. However, surgical intervention is preferred for patients with severe symptoms or those who do not respond adequately to medical management. Thoracic endovascular aortic repair (TEVAR) combined with revascularization presents a viable therapeutic option for symptomatic non-aneurysmal ARSA, potentially leading to favorable outcomes. It is essential to consider the indications and limitations of this minimally invasive approach, such as potential risks of left arm ischemia and basilar artery ischemia. Comprehensive preoperative assessment and diligent intraoperative monitoring can help minimize these risks, ensure optimal patient outcomes, and determine the appropriateness of this approach for each patient.

## References

[1] Kau T, Sinzig M, Gasser J (2007). Aortic development and anomalies. Semin Intervent Radiol.

[2] Janssen M, Baggen MGA, Veen HF (2000). Dysphagia lusoria: clinical aspects, manometric findings, diagnosis, and therapy. Am J Gastroenterol.

[3] Layton KF, Kallmes DF, Cloft HJ, Lindell EP, Cox VS (2006). Bovine aortic arch variant in humans: clarification of a common misnomer. AJNR Am J Neuroradiol.

[4] Polguj M, Chrzanowski Ł, Kasprzak JD, Stefańczyk L, Topol M, Majos A (2014). The aberrant right subclavian artery (arteria lusoria): the morphological and clinical aspects of one of the most important variations--a systematic study of 141 reports. ScientificWorldJournal.

[5] Levitt B, Richter JE (2007). Dysphagia lusoria: a comprehensive review. Dis Esophagus.

[6] Fanelli U, Iannarella R, Meoli A, Gismondi P, Cella S, Vincenzi F, Esposito S (2020). An unusual dysphagia for solids in a 17-year-old girl due to a lusoria artery: a case report and review of the literature. Int J Environ Res Public Health.

[7] Kieffer E, Bahnini A, Koskas F (1994). Aberrant subclavian artery: surgical treatment in thirty-three adult patients. J Vasc Surg.

[8] Gross RE (1946). Surgical treatment for dysphagia lusoria. Ann Surg.

[9] Bayford D (1794). An account of a singular case of obstructed deglutition. Memoirs Med Soc London.

[10] Tanaka A, Milner R, Ota T (2015). Kommerell's diverticulum in the current era: a comprehensive review. Gen Thorac Cardiovasc Surg.

[11] Edwards JE (1948). Anomalies of the derivatives of the aortic arch system. Med Clin North Am.

[12] Momma K, Matsuoka R, Takao A (1999). Aortic arch anomalies associated with chromosome 22q11 deletion (CATCH 22). Pediatr Cardiol.

[13] Backer CL, Mavroudis C, Rigsby CK, Holinger LD (2005). Trends in vascular ring surgery. J Thorac Cardiovasc Surg.

[14] An KR, Deng MX, Freud LR, Honjo O (2023). Repair of aberrant right subclavian artery causing dysphagia lusoria via partial median sternotomy. World J Pediatr Congenit Heart Surg.

